# Biliary Drainage and Complex Hepatobiliary Surgery in Biliary Tract Cancers: A Framework for Selective Centralization

**DOI:** 10.3390/cancers18213572

**Published:** 2026-11-06

**Authors:** Dennis Björk, Emine Selda Gündüz, Piotr Kalinowski, Giovanni Scotton, Rocio I. R. Macias, Elene Mariamidze, Anna Saborowski, Gonzalo Sapisochin, Angela Lamarca

**Affiliations:** 1 Department of Biomedical and Clinical Sciences, Linköping University, 581 83 Linköping, Sweden; dennis.bjork@liu.se; 2 Clinical Department of Surgery in Linköping, Region Östergötland, 581 83 Linköping, Sweden; 3 Department of Medical Services and Techniques, Vocational School of Health Services, Akdeniz University, Antalya 07070, Türkiye; seldagunduz@akdeniz.edu.tr; 4 Department of General, Transplant and Liver Surgery, Medical University of Warsaw, 02-091 Warsaw, Poland; piotr.kalinowski@wum.edu.pl; 5 Department of General and Visceral Surgery, Academic Hepatobiliary Center, Kepler University Hospital, 4021 Linz, Austria; giovanni.scotton@kepleruniklinikum.at; 6 Experimental Hepatology and Drug Targeting (HEVEPHARM) Group, University of Salamanca, IBSAL, 37007 Salamanca, Spain; rociorm@usal.es; 7 Centro de Investigación Biomédica en Red de Enfermedades Hepáticas y Digestivas (CIBERehd), 28029 Madrid, Spain; 8 Department of Oncology and Hematology, Todua Clinic, 0112 Tbilisi, Georgia; elenemariamidze01@gmail.com; 9 Department of Gastroenterology, Hepatology, Infectious Diseases and Endocrinology, Hannover Medical School, 30625 Hannover, Germany; saborowski.anna@mh-hannover.de; 10 Department of Abdominal, General Surgery and Liver Transplantaion, Hepatobiliary and Liver Transplant Unit, Hospital Universitario Vall d’Hebron, CIRCATH Group, Vall d’Hebron Institute of Research (VHIR), 08035 Barcelona, Spain; gonzalo.sapisochin@vallhebron.cat; 11 Department of Medical Oncology, OECI Comprehensive Cancer Centre—ENETS Centre of Excellence (GEP and Lung)—Fundacion Jimenez Diaz University Hospital, Health Research Institute Fundación Jiménez Díaz (IIS-FJD), Universidad Autónoma de Madrid (UAM), 28049 Madrid, Spain

**Keywords:** biliary tract cancer, cholangiocarcinoma, biliary drainage, hepatobiliary surgery, liver transplantation, future liver remnant, portal vein embolization, centralization of care

## Abstract

**Background/Objectives**: Biliary tract cancers (BTCs) are rare, heterogeneous malignancies that frequently present with biliary obstruction and require complex multidisciplinary care. Decisions regarding biliary drainage, future liver remnant (FLR) optimization, and major hepatobiliary surgery may influence perioperative risk and treatment eligibility. This narrative review evaluates the evidence supporting selective centralization and proposes a network-based framework that considers tumor subtype, procedural complexity, and access to care. **Methods**: The scope and principal topics were defined during the multidisciplinary COST Training School “Biliary Tract Cancers: Practical Approaches in Challenging Environments”, held in Barcelona, Spain, on 20–21 March 2026. Members of the author group conducted topic-specific PubMed/MEDLINE searches within their areas of expertise. Guidelines, systematic reviews, meta-analyses, and relevant clinical studies were synthesized narratively. BTC-specific findings were distinguished from evidence derived from mixed populations. No formal risk-of-bias assessment or consensus voting process was undertaken. **Results**: Preoperative biliary drainage should be selective, with the indication, target, and modality determined by clinical status, biliary anatomy, treatment intent, and planned intervention. FLR assessment should integrate volume and function, particularly in patients with cholestasis or cholangitis. Evidence supporting augmentation strategies varies by technique and population. Observational studies associate higher procedural volumes with favorable outcomes, although patient selection and case-mix differences limit causal interpretation. Tumor-specific pathways distinguish perihilar, intrahepatic, and distal cholangiocarcinoma and gallbladder cancer. The proposed network combines expert-center coordination of complex interventions with regional care supported by shared protocols, defined responsibilities, escalation pathways, and outcome monitoring. **Conclusions**: Selective centralization offers an organizational approach to balancing specialist expertise with timely and equitable access. Prospective multicenter evaluation is needed to assess safety, treatment delays, access to curative therapy, patient-centered outcomes, and resource use before wider clinical or policy adoption.

## Simple Summary

Biliary tract cancers are uncommon and aggressive tumors that often cause blockage of the bile ducts. Decisions regarding how to drain the bile ducts, how to prepare the liver for major resection, and where surgery should be performed can strongly influence outcome. The topics of this review were selected and debated during a workshop, held in Barcelona, Spain, in March 2026, addressing priorities for the centralization of care in biliary tract malignancies. This review examines the evidence supporting care in experienced centers and considers the disadvantages of concentrating treatment in a small number of hospitals. We propose selective centralization: complex biliary drainage, major hepatobiliary surgery, transplant assessment, and management of complications should be coordinated or performed in expert centers, while suitable elements of assessment, recovery, and follow-up may be delivered closer to home. This model aims to combine specialist expertise with timely, equitable, and continuous care for patients with biliary tract cancer.

## 1. Introduction

Biliary tract cancers (BTCs) make up a rare and heterogeneous group of malignancies arising from the biliary epithelium, including intrahepatic (iCCA), perihilar (pCCA), and distal cholangiocarcinoma (dCCA), as well as gallbladder cancer (GBC). These tumors differ substantially in their anatomical presentation, biological behavior, patterns of spread, and therapeutic options. Nevertheless, they share an aggressive biological behavior, frequent diagnosis at an advanced stage, and limited eligibility for potentially curative treatment. Surgical resection remains the principal curative-intent treatment for localized disease, while liver transplantation may be considered in highly selected patients, particularly those with unresectable pCCA and iCCA treated within established protocols [1,2,3,4,5].

The management of BTC is especially challenging because diagnostic and therapeutic decisions are closely interdependent. High-quality cross-sectional imaging and accurate assessment of biliary and vascular anatomy are required before any intervention. In patients presenting with biliary obstruction, the indication, timing, target, and modality of drainage may influence diagnostic accuracy, the risk of cholangitis, the feasibility of future liver remnant (FLR) optimization, and subsequent surgical eligibility. Biliary interventions may require endoscopic retrograde cholangiopancreatography (ERCP), percutaneous transhepatic biliary drainage (PTBD), or, in selected situations, endoscopic ultrasound (EUS)-guided techniques. The optimal approach varies according to tumor location, ductal anatomy, treatment intent, previous interventions, and local expertise. Inappropriate or incomplete drainage may result in infectious complications, repeated procedures, delays in treatment, or loss of eligibility for curative-intent strategies [2,6,7,8].

Complex hepatobiliary surgery introduces additional challenges. Resection of pCCA generally requires extended hepatectomy, caudate lobectomy, extrahepatic bile duct resection, regional lymphadenectomy, biliary reconstruction, and, in selected patients, portal vein or hepatic artery resection and reconstruction. Similarly, selected patients with iCCA or GBC generally require major liver resection, complex lymphadenectomy, or multivisceral procedures. The safety of these operations depends not only on technical resectability but also on the volume and function of the FLR. Preoperative planning therefore includes volumetric and functional liver assessment followed by staged strategies intended to reduce the risk of post-hepatectomy liver failure. Staged procedures are based on a concept of augmentation of the volume and function of the FLR and include portal vein embolization (PVE), combined portal and hepatic vein embolization (PVE/HVE), or a surgical procedure of associating liver partition and portal vein ligation for staged hepatectomy (ALPPS). After successful augmentation of the FLR the final procedure is a major hepatectomy [9,10,11].

These interventions require coordinated input from hepatobiliary surgeons, interventional endoscopists, interventional radiologists, diagnostic radiologists, hepatologists, oncologists, pathologists, transplant teams, anesthesiologists, intensive care specialists, and specialized nursing staff. Consequently, outcomes may depend not only on the individual procedure but also on institutional experience, multidisciplinary coordination, perioperative infrastructure, and the capacity to recognize and manage complications. Observational studies, population-based analyses, and systematic reviews have demonstrated associations between higher hospital or surgeon volume and lower perioperative mortality, improved failure-to-rescue rates, more frequent complete oncological resection, and better long-term outcomes after complex hepatobiliary surgery [12,13].

However, centralization is not without limitations. Requiring all diagnostic procedures, interventions, treatment, and follow-up to occur at a small number of tertiary centers may increase travel burden, delay urgent care, create capacity constraints, and widen geographical or socioeconomic disparities [6,14]. Moreover, most of the evidence supporting centralization is observational and may be influenced by patient selection, referral patterns, and differences in institutional resources [15]. The relevant question is therefore not whether all BTC care should be centralized, but which decisions and interventions require expert-center coordination, which components can be safely delivered within regional networks, and what organizational safeguards are necessary to maintain quality and continuity. A selective model may centralize complex decision-making, high-risk biliary interventions, liver augmentation, major hepatobiliary surgery, transplant assessment, and rescue care, while allowing appropriate elements of initial evaluation, postoperative recovery, and long-term follow-up to be provided closer to the patient [16].

This narrative review examines the evidence regarding biliary drainage, FLR assessment and augmentation, complex hepatobiliary surgery, transplantation assessment, and perioperative rescue pathways in patients with BTC. It also evaluates the volume–outcome relationship, the potential benefits and limitations of centralization, and the organizational requirements for a tiered hub-and-spoke network. Based on the available evidence and multidisciplinary expert interpretation, we propose a framework for selective centralization that aims to balance procedural expertise, timely access, equity, and continuity of care. Systemic anticancer therapy is beyond the scope of this review, except where multidisciplinary coordination affects the timing or sequencing of biliary and surgical interventions.

## 2. Methods

### 2.1. Scope Definition

The scope and principal topics of this narrative review were defined during the multidisciplinary COST Training School “Biliary Tract Cancers: Practical Approaches in Challenging Environments”, held in Barcelona, Spain, on 20–21 March 2026. Workshop discussions identified the main clinical domains addressed in the review. No formal Delphi process, voting procedure, or predefined consensus threshold was used.

### 2.2. Literature Search and Evidence Selection

This article was developed as a narrative review. Members of the multidisciplinary author group conducted topic-specific searches of PubMed/MEDLINE within their respective areas of expertise, covering the literature from database inception to 19 July 2026. Search terms and combinations varied across sections according to the clinical and organizational questions addressed and covered biliary tract cancer, biliary drainage, future liver remnant assessment and augmentation, complex hepatobiliary surgery, liver transplantation, perioperative outcomes, procedural volume, centralization, regionalization, and hub-and-spoke models. Priority was given to clinical guidelines, systematic reviews, meta-analyses, prospective studies, large multicenter or population-based cohorts, and benchmark studies. Selected mixed hepatobiliary or liver-resection studies were included when disease-specific evidence was limited and were interpreted as indirect evidence. Reference lists of relevant publications were also reviewed. Relevant studies were selected and interpreted by members of the author group according to their clinical and methodological expertise. No formal systematic review protocol, risk-of-bias assessment, or quantitative synthesis was undertaken. These methodological limitations should be considered when interpreting the conclusions and the proposed selective centralization framework.

### 2.3. Evidence Synthesis and Framework Development

Evidence was synthesized thematically across the predefined clinical domains. The proposed hub-and-spoke framework was developed by integrating the literature with the clinical and organizational issues identified during the workshop and was subsequently reviewed by the author group. It should be interpreted as a conceptual model for future evaluation rather than a formally validated clinical guideline or consensus statement.

The evidence is discussed in relation to study design, patient population, tumor subtype, treatment intent, and outcome definitions. Where findings differ, these contextual differences and the limitations of individual studies are highlighted. BTC-specific evidence is distinguished from findings derived from mixed populations, and unresolved findings are presented as context-dependent rather than as a basis for definitive recommendations. No standardized risk-of-bias assessment was performed.

Clinical recommendations attributed to published guidelines retain their original evidentiary basis and should be distinguished from the organizational arrangements proposed by the authors. The workshop informed the selection of topics and discussion of care priorities; it was not intended to establish formal consensus recommendations. The proposed framework brings together the reviewed evidence and multidisciplinary perspectives to inform service organization, while allowing adaptation to regional resources and referral pathways.

## 3. Biliary Drainage in the Multidisciplinary Management of BTC

### 3.1. Indications, Timing, and Drainage Targets

Biliary obstruction is a common clinical presentation in patients with BTC, particularly in patients with pCCA and dCCA. It may present with elevated plasma bilirubin or jaundice, cholestasis, pruritus, cholangitis, and/or impaired liver function. Biliary drainage may be required to control infection, relieve severe cholestasis, restore hepatic function, or prepare selected patients for FLR augmentation or major liver resection.

Whenever clinically feasible, high-quality cross-sectional imaging should be completed before biliary intervention. Endoscopic or percutaneous manipulation of the biliary tree may modify ductal anatomy, introduce infection, or complicate subsequent radiological assessment of tumor extent and resectability. Accordingly, the indication, target ducts, and intended drainage route should ideally be discussed within a multidisciplinary team before elective intervention [1,7,8].

The 2025 EASL guideline recommends avoiding routine preoperative biliary drainage in patients with resectable extrahepatic cholangiocarcinoma. It recommends considering drainage in the presence of cholangitis, renal failure, intractable pruritus, high bilirubin values, planned neoadjuvant chemotherapy, extensive surgery, or an anticipated prolonged wait for surgery [7]. These guideline recommendations should be distinguished from the heterogeneous findings of individual studies evaluating associations between drainage and postoperative outcomes [17,18,19,20,21,22,23,24,25]. The indication for drainage should therefore be established in relation to the patient’s clinical condition and planned treatment.

For patients being considered for major hepatectomy or FLR augmentation, this assessment should additionally document the planned extent of resection, FLR volume and function, and whether the intended remnant is obstructed or inadequately drained. These findings should be reviewed alongside infection status, renal function, bilirubin levels, symptoms, and the anticipated treatment interval. The purpose is to establish the clinical need and anatomical target for drainage, rather than to apply a single bilirubin cutoff or require drainage solely because augmentation is planned [7].

In pCCA requiring major hepatectomy, drainage planning should focus on the segments intended to constitute the FLR rather than automatically aiming to drain the entire liver. This planning principle should not be interpreted as evidence that unilateral drainage is universally preferable to bilateral drainage. The appropriate extent of drainage depends on the planned resection, ductal anatomy, and the location of infected or inadequately drained segments. Observational findings concerning selective FLR drainage are discussed below [26].

### 3.2. Selection of Drainage Modality

Endoscopic retrograde cholangiopancreatography (ERCP), percutaneous transhepatic biliary drainage (PTBD), and endoscopic ultrasound-guided biliary drainage (EUS-BD) provide complementary rather than universally competing approaches. Current evidence does not demonstrate the superiority of a single modality across all anatomical and clinical settings. The choice should be individualized according to tumor location, ductal anatomy, treatment intent, previous interventions, urgency, transplant considerations, and institutional expertise [27,28,29].

For patients with resectable dCCA who have an indication for biliary decompression, the 2025 EASL guideline recommends endoscopic rather than percutaneous drainage [7]. A propensity score-matched observational study reported less favorable oncological outcomes following PTBD than endoscopic drainage [30]; however, this association does not establish causality or exclude residual confounding. For resectable pCCA requiring drainage, the same guideline does not recommend endoscopic over percutaneous drainage because comparative evidence is insufficient [7]. The drainage route should therefore be selected according to the anatomical and clinical setting, treatment intent, and available expertise.

The management of hilar obstruction is more complex. ERCP may allow internal drainage but can be technically challenging in high-grade strictures. Contrast opacification of ducts that cannot subsequently be drained may increase the risk of cholangitis. PTBD may facilitate selective access to specific liver segments and can be advantageous when precise drainage of the intended FLR is required. However, it is associated with external catheter-related complications, including bleeding, dislodgement, patient discomfort, and the potential risk of tract-related tumor seeding. The risks and benefits of endoscopic and percutaneous access should therefore be assessed on an individual basis rather than according to a rigid location-based algorithm.

The route of drainage may also influence subsequent treatment options. In patients who may be candidates for liver transplantation, invasive diagnostic or biliary procedures should be discussed with the transplant center before intervention whenever the clinical situation permits. Percutaneous procedures may carry a risk of tumor dissemination and could affect eligibility for transplantation under certain protocols. Importantly, percutaneous biopsy of the tumor is a formal contraindication for liver transplantation. Emergency stabilization and drainage of life-threatening cholangitis should not, however, be delayed solely to complete a tertiary-center assessment.

EUS-BD is increasingly used as an alternative after failed or unfeasible ERCP, particularly in patients with distal malignant biliary obstruction. Techniques such as EUS-guided choledochoduodenostomy or hepaticogastrostomy may provide effective internal drainage in experienced centers [31]. Electrocautery-enhanced lumen-apposing metal stents (EC-LAMS) represent a technical development in therapeutic EUS. Mangiavillano et al. reported a retrospective multicenter series of 58 patients undergoing various EUS-guided interventions, including 22 patients receiving biliary drainage for malignant distal obstruction [32]. The study reported favorable technical and clinical outcomes, but its mixed indications and retrospective design limit extrapolation to BTC-specific treatment pathways.

Nevertheless, their role in potentially resectable disease and complex hilar obstruction remains more selective. These procedures should be performed by teams with expertise in advanced therapeutic endoscopy and immediate access to interventional radiology and surgical rescue.

The success of biliary drainage should not be evaluated solely according to technical stent or catheter placement. Clinically relevant outcomes include resolution of cholangitis, reduction in bilirubin concentration, adequate drainage of the intended FLR, avoidance of undrained opacified segments, procedure-related adverse events, the need for repeated interventions, and the time required to proceed to definitive treatment. Repeated unsuccessful procedures may delay surgery, promote infection with multidrug-resistant organisms, impair nutritional and functional status, and ultimately compromise eligibility for curative-intent treatment.

Evidence concerning drainage above versus across the ampulla remains context-dependent. A single-center prospective study reported in a conference abstract evaluated primary percutaneous metal stenting above the ampulla in patients with unresectable malignant hilar obstruction, using a historical endoscopic drainage cohort for comparison [33]. The findings are relevant to palliative drainage but do not establish a preferred preoperative approach for resectable iCCA or pCCA. The nonrandomized design and historical comparator also limit causal interpretation.

Similarly, a small observational subgroup analysis of patients with pCCA undergoing PVE found that unilateral drainage of the intended FLR was associated with greater subsequent hypertrophy than bilateral drainage [26]. This finding concerns a surrogate outcome in a selected population and does not establish superiority for postoperative morbidity, mortality, or all clinical settings. Decisions concerning unilateral or bilateral drainage should therefore remain individualized, particularly when infection or inadequate drainage affects additional liver segments.

### 3.3. Implications for Selective Centralization

The complexity of biliary drainage varies considerably among patients. Emergency stabilization, antibiotic treatment, and urgent decompression should not be delayed solely to arrange transfer to a tertiary institution. Selected ERCP or PTBD procedures may be performed in regional centers when appropriately trained teams, standardized protocols, and rapid consultation and referral pathways are available.

Conversely, suspected pCCA, complex hilar anatomy, planned selective FLR drainage, recurrent cholangitis, failed or incomplete initial drainage, the need for EUS-guided intervention, and possible transplant candidacy should prompt early discussion with an expert center. In these settings, an inappropriate initial intervention may alter the biliary anatomy, complicate or hinder adequate radiological image interpretation, introduce infection, increase the number of subsequent procedures, or adversely affect eligibility for curative-intent treatment.

The appropriate level of centralization should therefore be determined by the complexity and potential consequences of the planned intervention. Centralization is particularly relevant for defining the drainage strategy, performing technically demanding interventions, and managing complications or failed procedures. By contrast, selected routine procedures, emergency decompression when transfer would be unsafe, and subsequent stent or catheter care may be delivered regionally by credentialed teams.

The British Society of Gastroenterology guideline recommends clearly established pathways for patients presenting with jaundice or biliary obstruction, streamlined access to local and regional hepatopancreatobiliary multidisciplinary teams, and defined monitoring pathways after biliary stenting [34]. Building on these recommendations and published principles of surgical centralization [35], we propose that, within a tiered hub-and-spoke network, the expert multidisciplinary team coordinate the treatment sequence, drainage target, and access route when an intervention may influence resectability, FLR optimization, transplant eligibility, or major surgery. Selected procedures and routine drain care may be delivered regionally where appropriate expertise, shared protocols, rapid re-referral pathways, and prospective quality monitoring are available. This allocation of responsibilities represents the authors’ organizational proposal rather than a validated distribution of services.

## 4. Future Liver Remnant Assessment and Liver Augmentation

### 4.1. Volumetric and Functional Assessment of the FLR

Curative-intent surgery for BTC may require major or extended hepatectomy. The feasibility and safety of resection are determined not only by whether the tumor can be technically removed but also by whether the FLR will provide sufficient postoperative function. An insufficient FLR increases the risk of post-hepatectomy liver failure (PHLF), a serious and much feared complication that represents a predominant cause of hepatectomy-related morbidity and mortality [36,37,38,39,40,41].

Conventional volumetric thresholds have generally consider an FLR of at least 25% of the total functional liver volume sufficient in patients with a healthy liver, whereas thresholds of approximately 30% and 40% have been used in patients exposed to chemotherapy and those with chronic liver disease, respectively [42]. However, these thresholds should be regarded as approximate clinical references rather than absolute criteria. A large single-center study including 1748 patients proposed more conservative minimum FLR volumes of ≥30% for patients with unaffected liver parenchyma and ≥50% in those with impaired liver function [43]. These findings emphasize the importance of interpreting FLR volume in conjunction with parenchymal quality and functional assessment [42,43]. The reported percentages should be regarded as clinical reference values rather than uniformly validated safety thresholds for BTC. In patients with cholestasis or cholangitis, an apparently adequate FLR volume alone does not establish sufficient functional reserve; drainage status, infection control, and regional liver function must also inform the assessment.

Volumetric assessment alone is not sufficient in patients with biliary obstruction, cholangitis, prolonged cholestasis, or underlying liver disease, in whom functional impairment may be heterogeneous across the liver. Global liver functional evaluation is therefore recommended as a complementary approach using the indocyanine green (ICG) clearance test [44,45] or the liver function maximum capacity (LiMAx) test [46]. However, these methods do not directly quantify the function of the FLR.

Hepatobiliary scintigraphy with ^99m^Tc-mebrofenin has shown potential [47,48] but may not be readily available. Magnetic resonance imaging (MRI) with hepatocyte-specific contrast agents is frequently used in preoperative workup of BTCs and can, via the hepatic uptake index (HUI), assess both global and segmental liver function [49]. Combining MRI with measurements of FLR and calculation of HUI may serve as a practical and reliable tool without the need for additional evaluation tests. Nevertheless, the availability, methodology, and validated thresholds of functional tests vary among centers, and no single approach has been universally adopted.

The volumetric thresholds and functional assessment methods discussed above should not be interpreted as uniformly validated criteria across BTC subtypes. Their applicability should be considered in relation to the population studied, particularly whether patients with biliary obstruction or cholangitis were included.

The evaluation of surgical eligibility should therefore integrate anatomical resectability, FLR volume, regional liver function, biliary drainage status, parenchymal quality, patient comorbidity, and the anticipated complexity of the planned resection. Decisions should not be based on an isolated volumetric threshold.

### 4.2. Strategies for FLR Augmentation

When the predicted FLR is considered insufficient, preoperative liver augmentation may reduce the risk of PHLF and allow selected patients to undergo potentially curative resection. Portal vein embolization (PVE) remains the most established strategy. By occluding portal venous inflow to the liver segments scheduled for resection, PVE redirects portal flow toward the FLR and induces compensatory hypertrophy [50].

The response to PVE should be evaluated using both the absolute and relative increases in FLR volume and the rate of hypertrophy over time. Insufficient hypertrophy may indicate limited regenerative capacity and may identify patients at increased risk for PHLF after hepatectomy. The interval between PVE and surgery also provides an opportunity to reassess tumor biology and detect disease progression that may preclude resection.

The combination of PVE and hepatic vein embolization (PVE/HVE), also referred to as liver venous deprivation in some reports, has been shown to induce greater and more rapid hypertrophy than PVE alone [51,52,53,54]. The evidence supporting conventional PVE should be distinguished from that supporting combined PVE/HVE. BTC-specific experience includes reports of PVE in hilar cholangiocarcinoma and combined portal and hepatic vein embolization in pCCA [9,10]. Nevertheless, comparative findings from colorectal liver metastases or mixed liver-resection cohorts cannot be assumed to apply uniformly to BTC. In particular, greater or faster hypertrophy does not by itself establish improved postoperative safety or oncological outcomes. In patients with biliary obstruction or cholangitis, selection should therefore incorporate remnant liver function, drainage adequacy, and infection status, alongside volumetric response. The DRAGON PLC Trial registered at ClinicalTrials.gov is under way and is specifically studying primary liver malignancies in the setting of PVE/HVE [55].

Associating liver partition and portal vein ligation for staged hepatectomy (ALPPS) can produce rapid FLR hypertrophy and may permit resection in patients with a very small initial remnant [56]. However, the procedure is highly invasive and has been associated with substantial morbidity and mortality, particularly in patients with perihilar malignancies, impaired liver function, or biliary infection [57,58]. Its use in BTC should therefore remain highly selective and be restricted to expert centers after multidisciplinary evaluation. The expected oncological benefit must be carefully balanced against the procedural risk and the possibility of alternative augmentation strategies.

The selection between PVE, combined portal and hepatic vein embolization, staged surgery, or avoidance of resection should be individualized. Relevant factors include the initial FLR volume and function, the extent of the planned hepatectomy, biliary anatomy, the presence of cholangitis, tumor biology, vascular involvement, previous treatment, and institutional experience.

### 4.3. Biliary Drainage and the Sequencing of Liver Augmentation

Biliary drainage has traditionally been performed before liver augmentation in patients with obstructive jaundice, based on concerns that cholestasis may impair liver regeneration and increase the risk of procedural or postoperative complications. However, recent retrospective studies suggest that hyperbilirubinemia at the time of PVE does not negatively affect liver hypertrophy after PVE [26,59,60,61]. These findings challenge the assumption that normalization of bilirubin is an absolute prerequisite for PVE.

Nevertheless, the observation that hypertrophy can occur in the presence of hyperbilirubinemia should not be interpreted as evidence that biliary drainage is unnecessary. Cholangitis, impaired function of the intended FLR, persistent clinical deterioration, or inadequately drained segments remain important indications for intervention. Furthermore, serum bilirubin is a global marker and may not accurately reflect the drainage or function of the specific segments intended to remain after resection.

The appropriate sequence of drainage and liver augmentation should therefore be determined individually. In selected patients, PVE may be performed before complete biochemical normalization, particularly when delaying augmentation could postpone definitive treatment.

The interval between biliary drainage and PVE was not significantly associated with PHLF, although longer intervals showed a trend toward increased complications. Therefore, PVE may be performed early after biliary drainage in clinically appropriate patients [62]. Shortening the interval may therefore be beneficial. Simultaneous PVE and PTBD has also been reported to be feasible and safe in experienced centers [60,61,63,64]. This approach may reduce the number of separate procedures and shorten the preoperative pathway, but it requires careful planning to minimize bleeding, infection, catheter-related complications, and interference between biliary and portal access routes. The evidence is mainly based on retrospective cohort studies and larger randomized prospective studies would be beneficial to clarify patient selection and timing.

The central multidisciplinary team should define which liver segments require drainage, which portal branches should be embolized, and whether the procedures should be sequential or combined. This planning is particularly important in pCCA, where the drainage target, the intended FLR, and the side of portal vein embolization are anatomically and functionally interdependent.

### 4.4. Implications for Selective Centralization

FLR evaluation and liver augmentation require more than the technical ability to perform portal vein embolization. They depend on expert hepatobiliary imaging, standardized volumetry, access to regional functional assessment, interventional radiology, complex surgical planning, and coordinated management of biliary obstruction and infection.

Standard PVE may be performed in regional centers with appropriate experience, infrastructure, and established collaboration with the surgical hub. However, patients with complex hilar anatomy, uncertain resectability, impaired or discordant FLR volume and function, previous unsuccessful augmentation, or a proposed combined portal and hepatic vein embolization or staged procedure should be discussed with and generally managed at an expert hepatobiliary center.

Postprocedural assessment should also be coordinated centrally when the results determine eligibility for major resection. This includes evaluation of FLR hypertrophy and function, exclusion of disease progression, reassessment of biliary drainage, and confirmation that the anticipated operative benefit remains justified.

Within a hub-and-spoke model, selected imaging and laboratory follow-up may be performed regionally, while the augmentation strategy, technical execution of complex procedures, interpretation of the response, and final decision to proceed to surgery should remain under expert multidisciplinary oversight. This approach supports timely access while concentrating the decisions and procedures most closely associated with the risk of PHLF and perioperative mortality.

## 5. Complex Hepatobiliary Surgery and Selected Transplant Pathways

### 5.1. Surgical Complexity and Oncological Principles

Radical surgical resection is the only potentially curative treatment option for patients with localized BTC [20]. However, the type and complexity of surgery vary substantially according to tumor location, anatomical extension, vascular involvement, lymph-node status, and the functional capacity of the FLR. Accurate preoperative staging and multidisciplinary assessment are therefore essential to determine whether a macroscopically and microscopically complete resection can be achieved with an acceptable perioperative risk [2,13,20].

The objective of surgery is an R0 resection combined with an oncologically appropriate regional lymphadenectomy. Nevertheless, the pursuit of negative margins should not result in an operation for which the predicted morbidity or mortality outweighs the expected oncological benefit. Surgical planning must integrate tumor biology, anatomical resectability, patient frailty and comorbidity, liver function, FLR volume and function, and the anticipated need for biliary or vascular reconstruction.

Adequate lymph-node assessment is important for staging, prognostication, and postoperative treatment planning. Although the required extent and prognostic effect of lymphadenectomy differ among BTC subtypes, omission or inadequate performance of regional lymphadenectomy may lead to understaging and compromise the oncological quality of the procedure [65,66,67]. This component of surgery may itself be technically demanding, particularly in patients with previous biliary interventions, inflammation, or tumor involvement of the hepatoduodenal ligament.

### 5.2. Tumor-Specific Surgical Considerations

Surgery for pCCA represents one of the most technically demanding procedures in hepatobiliary surgical oncology. Curative-intent treatment commonly requires extrahepatic bile duct resection combined with major or extended hepatectomy, caudate lobe resection, regional lymphadenectomy, and reconstruction of one or more intrahepatic bile ducts. Portal vein resection and reconstruction may be required when necessary to achieve complete tumor clearance, whereas hepatic artery resection remains more selective because of its technical complexity and associated risks. The final operative strategy should be determined only after integrating biliary and vascular anatomy, lobar atrophy or hypertrophy, FLR assessment, and the results of any preoperative drainage or augmentation procedure [13,20,68].

iCCA encompasses a broad spectrum of diseases. Surgical treatment may range from limited anatomical or non-anatomical resection to extended hepatectomy with vascular or multivisceral resection and/or reconstruction. The need for major resection should be balanced against the likelihood of achieving an R0 resection, the presence of multifocal or extrahepatic disease, regional lymph-node involvement, and the expected postoperative liver function. Regional lymphadenectomy should be incorporated into the oncological operation both for staging and because adequate nodal assessment may be associated with improved prognostic stratification [67].

Surgical management of GBC depends on the stage and circumstances of diagnosis. Patients with incidental disease may require re-resection of the gallbladder bed or adjacent liver parenchyma and regional lymphadenectomy. More advanced tumors may necessitate major hepatectomy, bile duct resection, or multivisceral resection, although extended procedures should be undertaken only when an R0 resection is realistically achievable and the expected oncological benefit justifies the increased operative risk [69,70].

dCCA is generally treated by pancreaticoduodenectomy rather than liver resection. Although its operative pathway differs from that of pCCA and iCCA, it similarly requires experienced pancreatic and hepatobiliary surgical teams, standardized lymphadenectomy, appropriate margin assessment, and specialized management of postoperative complications. The present review focuses primarily on biliary drainage and complex liver surgery, but distal disease remains relevant when drainage strategy and institutional expertise influence progression to curative-intent surgery.

Table 1 summarizes tumor subtype, principal decision pathways and expert-center referral considerations.

Minimally invasive liver surgery may reduce blood loss, postoperative morbidity, and length of stay in appropriately selected patients. However, the evidence cited in the present manuscript is not specific to BTC or to operations requiring complex hilar dissection and biliary or vascular reconstruction [71]. Minimally invasive techniques should therefore be considered an operative approach rather than an independent argument for centralization, and their use should depend on tumor anatomy, procedural complexity, and institutional expertise.

**Table 1:** Tumor-specific considerations for referral and selective centralization in biliary tract cancers.

Tumor Subtype	Principal Decision Pathway	Expert-Center Referral Considerations
pCCA [7]	Assess biliary and vascular anatomy, resectability, FLR volume and function, and the need for biliary drainage or liver augmentation before defining the operative strategy.	Complex hilar drainage; major or extended hepatectomy; biliary or vascular reconstruction; potential transplant eligibility.
iCCA [72]	Assess disease extent and the feasibility of liver resection; evaluate FLR requirements when major hepatectomy is anticipated.	Uncertain resectability; major hepatectomy with borderline FLR; vascular or multivisceral involvement; consideration of transplantation within an approved investigational pathway.
dCCA [7]	Follow a pancreatic surgical pathway centered on assessment for pancreaticoduodenectomy, with biliary drainage considered according to clinical indications.	Assessment and treatment by an experienced pancreatic surgical team with access to advanced endoscopy, interventional radiology, and postoperative rescue care.
GBC [70]	Determine disease stage and whether diagnosis was incidental; assess the need for and extent of definitive resection or re-resection.	Uncertain indications for re-resection; anticipated bile duct or major liver resection; multivisceral involvement; uncertain resection margins.

**Abbreviations**: dCCA, distal cholangiocarcinoma; FLR, future liver remnant; GBC, gallbladder cancer; iCCA, intrahepatic cholangiocarcinoma; pCCA, perihilar cholangiocarcinoma. Note: Clinical considerations are summarized from disease-specific guidelines [7,70,72]. The referral considerations represent the authors’ synthesis within the proposed selective centralization framework and should be adapted to individual clinical circumstances and regional expertise. They are not validated referral algorithms.

### 5.3. Perioperative Care and Failure to Rescue

The safety of complex BTC surgery depends not only on operative technique but also on the entire perioperative pathway. Preoperative optimization should include nutritional and functional assessment, treatment of infection, evaluation of cardiopulmonary risk, optimization of biliary drainage when indicated, and confirmation that FLR volume and function are adequate. Anesthetic management during major hepatectomy requires careful control of intravascular volume, bleeding, hepatic inflow and outflow, coagulation, temperature, and postoperative organ support.

Standardized enhanced recovery pathways can support earlier mobilization, enteral nutrition, removal of unnecessary drains or catheters, and shorter hospitalization without compromising safety in appropriately selected patients undergoing liver surgery [73]. Nevertheless, protocolization cannot replace individualized management in patients undergoing extended hepatectomy, vascular reconstruction, or complex biliary anastomosis.

Major postoperative complications include PHLF, bile leakage, intra-abdominal infection, hemorrhage, vascular thrombosis, cholangitis, renal or respiratory failure, and sepsis. Early recognition and timely intervention are critical because postoperative mortality is influenced not only by the incidence of complications but also by the institution’s ability to rescue patients after complications occur. Failure to rescue reflects the combined availability of experienced surgical teams, intensive care, interventional radiology, advanced endoscopy, diagnostic imaging, and emergency reoperation [74].

Complex BTC surgery should therefore be supported by around-the-clock access to appropriate diagnostic and therapeutic resources. Management may require percutaneous drainage, angiographic embolization, biliary intervention, portal or hepatic vascular procedures, relaparotomy, or prolonged organ support. The capacity to coordinate these interventions rapidly is a fundamental component of institutional quality and should be considered alongside procedural volume when defining an expert center.

Pathological assessment is also integral to surgical quality. Expert hepatobiliary pathology, standardized specimen handling, evaluation of bile duct and vascular margins, adequate lymph-node assessment, and access to intraoperative frozen-section analysis when clinically relevant contribute to accurate staging and subsequent treatment planning. Multidisciplinary review can lead to changes in diagnosis, resectability assessment, and therapeutic strategy and should remain central throughout the perioperative pathway [2,3,75].

### 5.4. Liver Transplantation in Selected Patients

Liver transplantation represents a potential curative-intent strategy for carefully selected patients with unresectable pCCA when performed within established protocols that combine rigorous staging, exclusion of metastatic or nodal disease, neoadjuvant treatment and strict selection criteria [76]. Because candidacy may be affected by previous diagnostic or biliary procedures, patients with potentially transplant-eligible disease should be discussed with a transplant center before percutaneous biopsy or transperitoneal intervention whenever the clinical situation permits.

Percutaneous procedures may carry a risk of tumor seeding and exclude transplantation under certain protocols [30,77]. This consideration must be balanced against the need for urgent biliary decompression or treatment of cholangitis. A potentially curative transplant pathway should not delay clinically necessary emergency care, but the route and target of intervention should be coordinated with the transplant team whenever feasible.

Liver transplantation for iCCA remains investigational for most patients. Emerging results in highly selected individuals, including patients with small tumors or disease controlled under specific protocols, are encouraging, but transplantation cannot currently be considered routine standard care for iCCA. Patients should preferably be evaluated within prospective clinical trials or formally approved institutional protocols [72,78].

Transplantation for BTC requires an additional level of centralization beyond that required for conventional liver surgery. Outcomes depend on experience with disease-specific selection, neoadjuvant pathways, transplant oncology, complex biliary malignancies, and long-term graft and oncological surveillance. Recent evidence suggesting better outcomes in more experienced transplant centers further supports concentration of these pathways in a limited number of specialized institutions [79].

### 5.5. Clinical Implications

The complexity of surgery for BTC should not be defined solely by the number of liver segments removed. Even a relatively limited resection may require demanding hilar dissection, regional lymphadenectomy, reconstruction of small intrahepatic bile ducts, or surgery in tissues affected by cholangitis and previous biliary interventions. Conversely, selected major hepatectomies may be performed safely when anatomical planning, FLR optimization, perioperative care, and rescue capabilities are appropriately coordinated.

Decisions regarding referral should therefore integrate tumor subtype and anatomical location, the anticipated extent of liver, bile duct, vascular, pancreatic, or multivisceral resection, the need for biliary or vascular reconstruction, FLR volume and function, previous drainage-related or infectious complications, potential transplant eligibility, and patient frailty and comorbidity. Institutional capacity to recognize and manage major postoperative complications should also be considered. Taken together, these factors provide a more clinically meaningful basis for referral to an expert center than procedural volume or the extent of hepatectomy alone and support the selective centralization of patients requiring complex surgical or perioperative pathways.

## 6. Evidence Supporting Selective Centralization

### 6.1. Volume–Outcome Relationships

Evidence from hepatobiliary surgery consistently shows an association between higher hospital and surgeon volume and improved perioperative outcomes. Early studies of surgical regionalization demonstrated lower operative mortality in institutions and among surgeons with greater procedural experience [80,81,82]. More recent systematic reviews and population-based analyses have similarly associated higher liver resection volume with lower postoperative mortality and, in some studies, shorter hospital stay [83,84]. These findings are particularly relevant to BTC, because the rarity of these tumors limits institutional and individual surgeon exposure to complex biliary and liver procedures.

The potential benefits of treatment at specialized centers extend beyond perioperative mortality. Facility-level and multicenter studies have reported associations between care at academic or high-volume centers and higher rates of complete oncological resection, improved patient selection, and better long-term outcomes in BTC and pCCA [3,6,13]. Expert radiological and multidisciplinary reassessment may identify patients who could benefit from biliary optimization, liver augmentation, transplantation assessment, or surgery, while avoiding high-risk procedures when complete resection is unlikely.

Nevertheless, procedural volume should be regarded primarily as a surrogate for accumulated experience and institutional organization. Definitions of high volume vary across studies, countries and healthcare systems, and no universally accepted numerical threshold defines an expert BTC center. Annual caseload should therefore be considered together with procedure-specific outcomes and the availability of appropriate multidisciplinary and perioperative resources [35].

### 6.2. Institutional Expertise and Rescue Capacity

An expert BTC center should be defined by coordinated capabilities rather than surgical volume alone. Relevant resources include specialist hepatobiliary radiology, advanced endoscopy, interventional radiology, liver volumetry and functional assessment, complex hepatobiliary surgery, intensive care, pathology, transplantation assessment, and specialized nursing. A dedicated multidisciplinary team is essential because decisions concerning biliary drainage, FLR optimization, resectability, and transplant eligibility are closely interdependent [2,3,75,85].

The capacity to recognize and manage complications is another major determinant of institutional quality. Postoperative mortality depends not only on the occurrence of complications but also on the ability to prevent death once a complication has developed. This concept, referred to as failure to rescue, reflects staffing, surveillance, communication, intensive care provision, interventional radiology, therapeutic endoscopy, and timely surgical reintervention [74]. Consequently, rescue capability may explain part of the observed volume–outcome relationship and should be considered when designating referral centers.

Assessment of institutional performance should therefore extend beyond case volume. Relevant indicators include major morbidity, 30- and 90-day mortality, post-hepatectomy liver failure, bile leakage, unplanned reinterventions, readmission, and failure-to-rescue rates. Prospective monitoring of these outcomes would allow referral policies to be based on demonstrated quality rather than institutional designation alone.

### 6.3. Interpretation of the Evidence

The evidence supporting centralization remains predominantly retrospective and observational. Comparisons between high- and low-volume institutions may be affected by differences in patient selection, disease stage, referral patterns, socioeconomic factors, and access to specialist treatment. In addition, many studies include heterogeneous liver diseases and procedures, and their findings cannot always be extrapolated directly to BTC or to operations involving complex biliary reconstruction.

In particular, the evidence summarized here does not establish that centralization itself increases R0 resection rates independently of patient selection, disease stage, and anatomical resectability. Associations with complete resection should therefore not be interpreted as demonstrating an independent causal effect of centralization.

The available evidence therefore does not establish that every component of BTC care must be delivered at a central institution. It more strongly supports expert-center coordination of complex decision-making, high-risk biliary interventions, liver augmentation, major hepatobiliary surgery, transplantation pathways, and rescue care. Selected diagnostic procedures, routine postoperative recovery, and follow-up may be provided regionally when shared protocols, credentialing, outcome monitoring, and rapid re-referral pathways are in place.

Overall, the evidence favors selective centralization based on procedural complexity, institutional capability, and audited outcomes, rather than indiscriminate transfer of all patients or reliance on volume thresholds alone. This interpretation provides the rationale for considering both the limitations of centralization and the potential role of coordinated regional care.

## 7. Limitations of Centralization and Opportunities for Selective Decentralization

Although centralization may improve access to specialized expertise, concentrating care in a limited number of tertiary centers can increase travel burden, delay referral, create capacity constraints, and widen geographical or socioeconomic disparities [6,14]. These limitations are particularly relevant for patients requiring urgent treatment of cholangitis or repeated biliary interventions. Moreover, the evidence supporting centralization is predominantly observational and may be influenced by patient selection and referral patterns [15].

Centralization should therefore not be interpreted as requiring all components of care to be delivered at the expert center. Initial assessment, emergency stabilization, selected procedures, postoperative recovery, routine follow-up, and supportive care may be provided regionally when teams operate within shared protocols and have rapid access to specialist consultation and re-referral. Evidence from mentored hub-and-spoke hepatobiliary networks suggests that selected activities can be decentralized when participating centers have appropriate expertise, infrastructure, multidisciplinary review, and prospective quality monitoring [16].

Within the proposed framework, referral decisions should consider both the expected benefit of specialist care and the additional time required to obtain it. Early image transfer and multidisciplinary review, together with regional completion of agreed investigations, may reduce avoidable duplication and travel [34]. When transfer would delay clinically necessary emergency treatment, stabilization and decompression should proceed locally with expert consultation where feasible. If referral introduces a substantial delay, the treatment plan should be reassessed rather than assuming that transfer remains beneficial. Network evaluation should record referral-to-treatment intervals, disease progression before treatment, and reasons for loss of eligibility for curative-intent therapy, alongside perioperative outcomes. These are proposed safeguards requiring prospective evaluation.

A tiered model may therefore offer the most appropriate balance between expertise and accessibility: complex decision-making and high-risk interventions should be centralized, while suitable elements of care are delivered closer to the patient within a coordinated network.

## 8. A Tiered Hub-And-Spoke Model for BTC Care

Based on the evidence reviewed, we propose a tiered hub-and-spoke model in which the location of care is determined by procedural complexity, clinical urgency, and institutional capability rather than by a uniform requirement for transfer. The central expert hub should coordinate multidisciplinary assessment and provide complex biliary interventions, FLR optimization, major hepatobiliary surgery, transplantation evaluation, and rescue care. Regional centers may undertake selected diagnostic and interventional procedures when appropriately credentialed, while local hospitals retain responsibility for emergency stabilization, routine monitoring, postoperative recovery, and follow-up.

The contribution of this framework lies in bringing together BTC subtype, procedural complexity, and the sequence of care to guide the proposed distribution of responsibilities across a referral network. Rather than introducing a new hub-and-spoke structure, it applies established organizational principles to interconnected decisions concerning biliary drainage, FLR optimization, definitive surgery, transplant evaluation, and rescue care. The tumor-specific pathways and care-domain table make these proposed responsibilities explicit while identifying elements that may be delivered regionally.

The allocation of care across local, regional, and expert centers in this framework reflects the authors’ interpretation of the available evidence and multidisciplinary discussion. The framework has not been prospectively validated, and the predominantly retrospective and observational evidence does not establish the effectiveness of this specific allocation of services. It should therefore be regarded as a proposed organizational approach requiring adaptation to regional resources and referral pathways, rather than a validated clinical guideline. Prospective evaluation is needed to determine its effects on procedural safety, treatment delays, access to care, and patient outcomes.

Effective shared care requires standardized imaging and referral protocols, rapid transfer of clinical information, predefined escalation criteria, and direct communication between local teams and the central multidisciplinary team. Emergency treatment should not be delayed when transfer is unsafe, but elective interventions that may affect resectability, liver augmentation, or transplant eligibility should preferably follow expert review.

**Figure 1:**
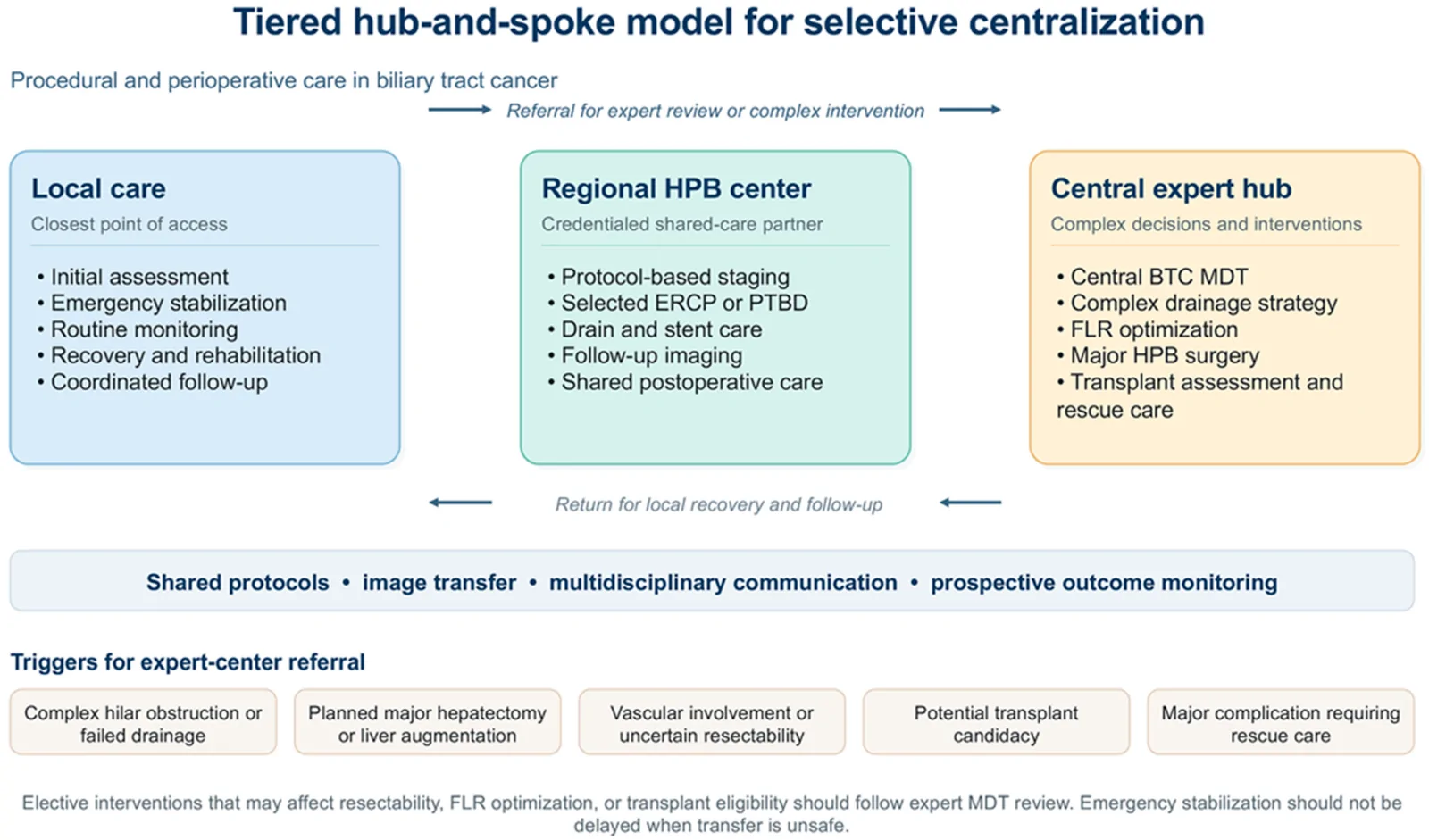
Priorities for centralization of care in biliary drainage and complex hepatobiliary surgery in biliary tract cancers: a framework for selective centralization. Tiered hub-and-spoke model for selective centralization of procedural and perioperative care in biliary tract cancer. The central expert hub coordinates multidisciplinary assessment and provides complex biliary interventions, future liver remnant optimization, major hepatobiliary surgery, transplantation assessment, and rescue care. Regional centers may undertake selected diagnostic and interventional procedures within formally coordinated pathways, while local hospitals provide emergency stabilization, routine monitoring, recovery, rehabilitation, and follow-up. Bidirectional referral and return-to-local-care pathways, shared protocols, rapid exchange of clinical information, and prospective quality monitoring are required to maintain continuity of care. Emergency treatment should not be delayed solely to arrange transfer. **Abbreviations**: BTC, biliary tract cancer; ERCP, endoscopic retrograde cholangiopancreatography; FLR, future liver remnant; HPB, hepatico-pancreatico-biliary; MDT, multidisciplinary team; PTBD, percutaneous transhepatic biliary drainage.

The proposed allocation of responsibilities and referral triggers is summarized in Table 2, while Figure 1 illustrates the bidirectional flow of patients between local, regional, and central levels of care. This framework is intended to support selective centralization while preserving timely and equitable access to appropriate care.

## 9. Organizational Requirements and Quality Indicators

Selective centralization requires defined referral pathways and access to multidisciplinary expertise [34]. Within the proposed network, written agreements should specify the coordinating team, information transfer, responsibility for drain care and follow-up, and urgent escalation arrangements. A joint governance group should oversee shared protocols, credentialing, and adverse-event review.

For each episode of regional care, the shared record should identify the responsible clinician, the agreed management and follow-up plan, and the route for urgent specialist advice. Escalation criteria should include clinical deterioration, persistent or recurrent cholangitis, failed or incomplete drainage, and complications exceeding local rescue capabilities. The network agreement should specify the receiving service and transfer arrangements, while joint audit should review delayed escalation, unplanned transfers, and adverse events. These safeguards are proposed requirements for regional delivery and require prospective evaluation.

Procedural volume is associated with outcomes after liver resection [84], but expert-center designation should also consider institutional capabilities and audited performance [35]. We propose procedure-specific credentialing based on training, experience, multidisciplinary support, and access to rescue services. Numerical volume thresholds and referral-time targets should be locally agreed and audited; they are not presented here as validated BTC-specific standards.

Prospective monitoring should include referral-to-review and treatment intervals, drainage success, reinterventions, complications, mortality, and failure to rescue. Building on established centralization principles [35], we additionally propose monitoring quality of life, continuity of care, travel and financial burden, and geographical equity. Funding agreements should cover coordination, shared care, and transfers. These organizational and reimbursement arrangements require adaptation to the healthcare system and prospective evaluation.

## 10. Future Directions

Future research should refine criteria for defining expert BTC centers, incorporating institutional capabilities and audited outcomes alongside procedural volume. Further evaluation should clarify the applicability of the proposed framework across BTC subtypes and healthcare systems, particularly where evidence derives from observational studies or mixed surgical populations. Procedure-specific referral criteria and operational targets should be adapted to regional resources and assessed prospectively.

Prospective multicenter registries and phased implementation studies could evaluate referral pathways, drainage strategies, liver augmentation, rescue care, and long-term outcomes. Comparisons with existing care pathways should account for tumor subtype, disease stage, patient risk, and procedural complexity. Relevant outcomes should include perioperative safety, treatment delays, disease progression before treatment, access to curative therapy, quality of life, travel burden, equity of access, resource use, and the safety of selected regional care.

Telemedicine, virtual multidisciplinary review, structured mentoring, and shared training programs may help extend specialist expertise while avoiding unnecessary patient transfer. Implementation should reflect differences in geography, workforce, infrastructure, and funding across healthcare systems. Findings from prospective evaluation, together with structured input from clinicians, patients, and healthcare commissioners, would support refinement of the framework and inform its suitability for wider clinical and policy adoption.

## 11. Conclusions

Biliary drainage, FLR optimization, and complex hepatobiliary surgery are closely interdependent components of BTC care. Their safe and effective delivery requires specialized technical expertise, multidisciplinary decision-making, appropriate perioperative infrastructure, and the capacity to rescue patients after major complications.

Current evidence supports selective rather than indiscriminate centralization. Complex drainage strategies, liver augmentation, major hepatobiliary surgery, transplantation assessment, and rescue care should be coordinated or performed in expert centers. Appropriate elements of assessment, postoperative recovery, and follow-up may be delivered regionally within structured networks. A tiered hub-and-spoke model may therefore provide a practical balance between expertise, timely access, equity, and continuity of care.

## Figures and Tables

**Table 2:** Proposed allocation of procedural and perioperative care for biliary tract cancer within a tiered hub-and-spoke network.

Care Domain	Local and Regional Network-Based Care	Central Expert Hub	Suggested Referral or Escalation Triggers
Initial assessment and staging	Clinical and laboratory assessment; stabilization of cholangitis or sepsis; protocol-based CT and/or MRI/MRCP; rapid transfer of images and clinical information	Expert hepatobiliary radiology review; assessment of resectability, vascular involvement, FLR, treatment sequencing, and transplant eligibility; central MDT recommendation	Suspected resectable pCCA; uncertain resectability; vascular involvement; borderline FLR; discordant or incomplete imaging
Biliary drainage	Emergency decompression when transfer would cause an unsafe delay; selected ERCP or PTBD by credentialed teams; routine stent and drain care	Drainage planning for complex hilar obstruction; selective drainage of the intended FLR; rescue after failed or incomplete drainage; advanced EUS-guided procedures	Complex hilar obstruction; recurrent cholangitis; failed or incomplete drainage; uncertain ductal anatomy; planned major hepatectomy or liver augmentation; potential transplant candidacy
FLR assessment and liver augmentation	Laboratory monitoring and protocol-based follow-up imaging after intervention	Integrated volumetric and functional assessment; selection and performance of PVE, combined portal and hepatic vein embolization, or selected staged strategies	Insufficient or borderline FLR; impaired liver function; planned extended hepatectomy; inadequate hypertrophy or clinical deterioration after augmentation
Complex hepatobiliary surgery	Prehabilitation, rehabilitation, and postoperative follow-up; selected lower-complexity procedures only in formally credentialed and audited network centers	pCCA surgery; major hepatectomy; biliary or vascular reconstruction; complex GBC re-resection; multivisceral procedures; surgical rescue	Hilar tumor; major hepatectomy; vascular involvement; complex biliary reconstruction; uncertain margins; need for multivisceral resection
Liver transplantation	Early identification and rapid referral; selected staging investigations under transplant-center guidance	Formal eligibility assessment; protocol-based neoadjuvant treatment where applicable; transplantation; registry or clinical-trial inclusion	Potentially transplant-eligible unresectable pCCA; highly selected iCCA considered within an approved protocol or clinical trial
Perioperative care and postoperative follow-up	Recovery after clinical stabilization; wound, drain, nutritional, and laboratory monitoring; rehabilitation and coordinated follow-up	Specialized perioperative pathways; intensive care; management of PHLF, bile leakage, hemorrhage, sepsis, and other major complications	Organ failure; uncontrolled bile leakage or hemorrhage; major infection; recurrent cholangitis; need for interventional, endoscopic, or surgical rescue
Coordination and quality assurance	Adherence to shared protocols; named care coordinator; timely information exchange; prospective outcome reporting	MDT leadership; training and credentialing; protocol development; benchmarking; morbidity and mortality review; network audit	Repeated procedural failure; excessive referral or treatment delays; failure to meet predefined quality indicators; unexpected morbidity or mortality signals

**Note**: This table represents the authors’ proposed framework derived from the evidence and multidisciplinary expert discussion presented in this review. Emergency stabilization and treatment of sepsis should not be delayed solely to arrange transfer. Whenever clinically feasible, elective interventions that may affect resectability, FLR optimization, or transplant eligibility should follow expert multidisciplinary review. Regional delivery of specialized procedures requires formal credentialing, shared protocols, prospective outcome monitoring, and predefined rapid re-referral pathways. **Abbreviations**: CT, computed tomography; ERCP, endoscopic retrograde cholangiopancreatography; EUS, endoscopic ultrasound; FLR, future liver remnant; GBC, gallbladder cancer; iCCA, intrahepatic cholangiocarcinoma; MDT, multidisciplinary team; MRCP, magnetic resonance cholangiopancreatography; MRI, magnetic resonance imaging; pCCA, perihilar cholangiocarcinoma; PHLF, post-hepatectomy liver failure; PTBD, percutaneous transhepatic biliary drainage; PVE, portal vein embolization.

## Data Availability

No new data were created or analyzed in this study. Data sharing is not applicable to this article.

## Abbreviations

The following abbreviations are used in this manuscript:

ALPPS	Associating liver partition and portal vein ligation for staged hepatectomy
BTC	Biliary tract cancer
CT	Computed tomography
dCCA	Distal cholangiocarcinoma
ERCP	Endoscopic retrograde cholangiopancreatography
EUS	Endoscopic ultrasound
EUS-BD	Endoscopic ultrasound-guided biliary drainage
FLR	Future liver remnant
GBC	Gallbladder cancer
HUI	Hepatic uptake index
HVE	Hepatic vein embolization
ICG	Indocyanine green
iCCA	Intrahepatic cholangiocarcinoma
LiMAx	Liver maximum capacity test
MDT	Multidisciplinary team
MRCP	Magnetic resonance cholangiopancreatography
MRI	Magnetic resonance imaging
pCCA	Perihilar cholangiocarcinoma
PHLF	Post-hepatectomy liver failure
PTBD	Percutaneous transhepatic biliary drainage
PVE	Portal vein embolization
